# Review of Foodborne Botulism in the UK: 2006–2024

**DOI:** 10.3390/foods14152584

**Published:** 2025-07-23

**Authors:** Corinne Francoise Laurence Amar, Burhan Ahmed, Jonathan Finch, Dunstan Rajendram, Vanessa K. Wong, Gauri Godbole

**Affiliations:** 1 Gastrointestinal Bacteria Reference Unit, UK Health Security Agency, 61 Colindale Avenue, London NW9 5EQ, UK; burhan.ahmed@ukhsa.gov.uk (B.A.); jonathan.finch@ukhsa.gov.uk (J.F.); dunstan.rajendram@ukhsa.gov.uk (D.R.); 2 Gastrointestinal Infections, Food Safety and One Health, UK Health Security Agency, London NW9 5EQ, UK; vanessa.wong@ukhsa.gov.uk (V.K.W.); gauri.godbole@ukhsa.gov.uk (G.G.)

**Keywords:** botulism, *Clostridium botulinum*, foodborne intoxication, botulism investigation, public health response

## Abstract

Food-borne botulism is a rare but serious disease caused by ingestion of botulinum neurotoxin pre-formed in food by *Clostridium botulinum.* Between 2006 and 2009, no foodborne botulism cases were reported in the UK. However, the period from 2010 to 2024 saw 13 cases, encompassing seven separate incidents and two outbreaks, with no reported fatalities. Cases were predominantly linked to imported, home-made, and artisanal foods, occasionally to commercial products. Diagnostic and public health challenges include delayed clinical diagnosis, delayed sample collection, inadequate specimen volumes, and the frequent unavailability of suspected food sources, hampering epidemiological investigations. The UK has an extremely low incidence of foodborne botulism with an estimated rate of 0.001 cases per 100,000 people per year, but despite this low occurrence, food botulism remains a public health emergency as it requires timely treatment and rapid reactive intervention to be undertaken by multiple regulatory agencies. Continuous professional training of medical staff, up-to-date clinical guidance, rapid diagnostic, and food investigations are essential for optimising patient outcomes and prevention.

## 1. Introduction

Botulism is a rare but life-threatening paralytic disease caused by a neurotoxin produced by *Clostridium botulinum*, an anaerobic, spore-forming bacterium. There are currently eight recognised botulinum neurotoxin (BoNT) types A to G and X, though only BoNT types A, B, E, and F are known to cause disease in humans [1]. In addition to *C. botulinum*, rare strains of *Clostridium butyricum* and *Clostridium baratii* can also produce BoNT E and BoNT F, respectively. A botulinum-like neurotoxin gene cluster has also been identified in a strain of *Enterococcus* sp. isolated from cow faeces, but its pathogenic potential remains uncertain [2].

Once in the bloodstream, BoNT binds to the neuromuscular junction, blocking the release of acetylcholine from synaptic vesicles and thereby preventing muscle contraction. This leads to a rapid, symmetrical, descending flaccid paralysis that can progress to respiratory failure, and without prompt intravenous administration of antitoxin, death may occur due to airway obstruction or respiratory arrest [3].

Foodborne botulism occurs when pre-formed BoNT is ingested through contaminated food or beverages in which *C. botulinum* has proliferated. Because the bacterium is a strict anaerobe, foods at highest risk include those stored in airtight conditions, such as canned goods, preserved foods in jars, and pickled products. The disease was first documented in the 18th and early 19th centuries in Germany, but its causative organism was identified in 1895 by the Belgian scientist Van Ermengem, following an outbreak linked to contaminated smoked ham at a funeral dinner [4]. In the UK, the first reported cases occurred in 1922, when eight individuals died within five days after consuming contaminated duck meat paste sandwiches [5].

A review published in 2006 documented 15 incidents of foodborne botulism in the UK between 1922 and 2005 [6]. Despite its rarity, the potential for respiratory failure and death of cases as well as the necessity of rapid source identification to prevent further exposure highlights the critical need for continued surveillance and understanding. For this effect, this review compiles and analyses the descriptive epidemiology of all recorded incidents and outbreaks of foodborne botulism in the UK from 2006 to 2024.

## 2. Materials and Methods

This review uses data from the established national surveillance and investigation framework detailed below in this section.

### 2.1. Case Identification and Data Collection

Suspected cases of botulism in England are initially identified through clinical diagnosis by healthcare professionals who notify potential cases to the Botulism Service at the UK Health Security Agency’s (UKHSA) and pre-2021 at its predecessors Public Health England and Health Protection Agency. The Botulism Service is a team of expert medical microbiologists and scientists who are providing expert and clinical advice and includes the UK reference laboratory for *C. botulinum* for testing. Botulism is a clinical diagnosis based on characteristic neurological symptoms resulting from BoNT activity at the neuromuscular junction. It presents with acute bilateral cranial nerve palsies, oculobulbar weakness, and a generalised, descending, symmetrical flaccid paralysis. Initially, patients typically report diplopia, dysarthria, dysphonia, dysphagia, and dry mouth. The affected patients remain afebrile and conscious with no sensory deficits, with constipation and urinary retention due to the progressive paralysis of autonomic smooth muscle. As the bilateral, descending muscle weakness worsens, the patients experience loss of head control, weakness of the trunk and upper limbs, and breathing difficulties, which ultimately leads to respiratory failure [3].

Relevant case details are documented in the UKHSA’s case and outbreak management system. This is an internal, secure system used by the clinicians and the Botulism service team which serves as the official data source for the cases reported in this review and where demographic details, initial symptoms, symptom progression, treatment, microbiology results, and outcomes are recorded. The local authority associated with the patient’s residence initiates an enhanced *C. botulinum* questionnaire for probable and confirmed cases. This involves a public health professional recording a comprehensive clinical history of the case(s) and completing a standardised food questionnaire, the later with a family member or, if the patient is well enough, with the patient themselves. This questionnaire aims to gather comprehensive food history, focusing on the consumption of high-risk foods in the seven days, then in the three days and the day preceding symptom onset.

### 2.2. Food Source Investigation and Data Analysis

Potential food items identified in the questionnaire are collected and sent to the reference laboratory for detection of botulinum neurotoxin or *C. botulinum*.

Implicated commercial, home-made, or artisanal products are notified to the UK Food Authority which initiates investigations into the product’s origin, potentially leading to the product retrieval from retailers and the issuing of national or international warning such as the Rapid Alert System for Food and Feed (RASFF). Data related to these investigations, including product details and public health actions, are incorporated into the outbreak management system. For home-made food, there are no broad epidemiological investigations or recall involved beyond the affected household.

The findings from these cases’ investigation, including clinical presentation, laboratory results, confirmed or suspected food sources and public health actions form the basis of the descriptive epidemiological data presented in this review and in Table 1. The classification of cases and the description of common theme, challenges, and outcomes are derived from these compiled surveillance records (outbreak management system and *C. botulinum* questionnaire) which are not publicly accessible due to patient confidentiality.

### 2.3. Microbiology Confirmation of Botulism

Clinical specimens, suspected food, and environmental samples are sent to the UK *C. botulinum* National Reference Laboratory for confirmation of the clinical diagnosis and identification of the food source responsible for intoxication.

Detection of BoNT is performed using a mouse bioassay (MBA), in which serum, faecal, or food extracts are injected intraperitoneally into the animals. Symptoms of botulism are observed up to 4 days, and confirmation, along with BoNT type identification, is carried out through a neutralisation test [7]. The presence of *C. botulinum* is confirmed by real-time PCR assays targeting the *BoNT* A, B, E, and F genes [8,9] and the organism is isolated using specialised media and enrichment broths [7].

## 3. Results

### 3.1. Number of Foodborne Botulism Cases Reported in the UK, 2006–2024

From 2006 to 2009, no cases were reported, but from 2010 to 2024, seven separate incidents and two outbreaks occurred totalling 13 cases without any fatalities. Descriptive epidemiological data available of all incidents are summarised in Table 1.

**Table 1:** Descriptive epidemiology summary of incidents and outbreaks of foodborne botulism in the UK between 2006 and 2024. Source data: UKHSA’s case and outbreak management system and case *C. botulinum* questionnaires (not publicly available) (PCR: polymerase chain reaction; MBA: mouse bioassay; ABE: *C. botulinum* anti-neurotoxin type A, B, and E pool).

Incident Date	Number of Cases	Food Source of Intoxication Confirmed (C) or Suspected (S)	*C. botulinum* Neurotoxin Type	Microbiology Confirmation of Clinical Diagnosis	Initial Treatment
Case July 2010	1	Acquired abroad, un-identified food served at wedding (S).	B	PCR from faeces (not recovered by culture).	Pneumonia.
Outbreak November 2011	3	Commercially distributed korma sauce in glass jar (C).	A	Serum MBA from 2 of the cases, PCR and culture from faeces of the 3 cases, MBA, PCR and culture from remnant of korma sauce, lid and emptied jar.	Delayed diagnosis in Case 1, botulism in Cases 2 and 3
Case July 2012	1	Olives in brine in glass jar, artisanal product from Italy (C).	B	PCR and culture from faeces, MBA, PCR and culture from olives in jar.	Botulism.
Case October 2013	1	Home-preserved mushroom in vinegar (S).	Not determined	MBA with ABE polyvalent neutralisation test from serum.	Gastroenteritis,
Case December 2016	1	Home-made tuna in oil (S).	B	PCR and culture from faeces, MBA from faeces.	Botulism
Case May 2019	1	Pizza topping (S).	Not determined	MBA with ABE polyvalent neutralisation test from serum.	Guillain Barre syndrome
Outbreak September 2023	3	Sardine in oil prepared by local restaurant (C).	B	PCR and culture from faeces from all 3 cases ^1^.	Botulism.
Case February 2024	1	Pub meal or home-made meat bakes (S).	B	MBA from serum, PCR and culture from faeces.	Botulism.
Case September 2024	1	Roasted chicken bites (S).	A	PCR and culture from faeces.	Stroke.

^1^ *C. botulinum* neurotoxin type B was detected in the sardines by the French reference laboratory [10].

### 3.2. Case Summary—July 2010

A male individual in his thirties travelled abroad to attend to a wedding. The day after arrival, he developed symptoms of food poisoning followed by neurological symptoms. He was discharged by the local hospital. Six days after symptom onset, he returned to the UK, was admitted to hospital with suspected pneumonia, and received antibiotics. His condition worsened and developed classical symptoms of botulism requiring intensive care support and was treated with botulinum antitoxin. He recovered and was discharged home a month later.

There was no history of consuming home-made or preserved food, and at the wedding, he ate vegetable soup, lamb meatballs, and roasted peppers, though their preparation method was unknown. The patient also mentioned consuming street food the day before falling ill without precising which types.

Serum was collected on UK hospital admission (six days after symptom onset) and faeces only seven days after his return due to severe constipation. *C. botulinum* type B was detected by PCR from faecal enrichment broths, but the organism could not be isolated and mouse bioassays from serum and faecal extracts were negative for BoNT. The final diagnosis was botulism, based on clinical symptoms and faecal PCR results. Since there were no food samples available for testing, a public health response was not initiated, as the suspected source was thought to be food consumed abroad, limiting further investigation within the UK.

### 3.3. Outbreak Summary—November 2011

In November 2011, on two consecutive days, the *C. botulinum* UK reference laboratory was alerted to two suspected botulism cases in siblings, aged 5 and 7 years. Both were clinically diagnosed with botulism, required ventilation, and received botulinum antitoxin. Their 3-year-old sister, initially asymptomatic, developed signs of botulism six days after the first child was admitted to hospital and was treated with antitoxin and discharged six days after admission [11].

A detailed food history covering three days before symptom onset identified potential sources and samples collected from the home’s refrigerator, cupboards, and recycling bins included several foods suspected based on biological plausibility. Amongst these were remnants of chicken korma which had been consumed two days before the first child became ill. These included the branded korma sauce with cooked chicken from the rubbish bin, an empty korma sauce jar and lid from the recycling bin, and an unopened jar of the same brand from the kitchen cupboard [11].

Mouse bioassay (MBA) of serum from the 5- and 7-year-olds showed classic botulism symptoms, but there was insufficient sample for neutralisation testing. *C. botulinum* type A was detected in the rectal washout of the 5-year-old and the faeces of the 7-year-old, though BoNT was not detected by MBA. The 3-year-old also tested positive for *C. botulinum* type A in faeces and her serum was untestable due to haemolysis and collection in EDTA, which does not meet UK Animals (Scientific Procedures) Act 1986 refinement obligations.

BoNT type A and *C. botulinum* type A were detected in the chicken/korma sauce remnants, the empty jar, and its lid. In contrast, the unopened jar from the same brand tested negative. The implicated korma sauce batch was subsequently withdrawn in the UK and Ireland, with warnings issued to consumers, medical professionals, and the public, and similar alerts were disseminated across Europe. Investigation into the growth of *C. botulinum* and subsequent production of neurotoxin in the Korma jar was undertaken but remained inconclusive [11]. Examination of loss of vacuum in two jars from the implicated batch showed they received impact damage on the lid which released vacuum. The hypothesis was that this could have raised the pH of the product and consequently allowed germination of spores in the implicated jar; however, this was never shown to be conclusive, and the cause of the Korma jar’s contamination remained unknown. Furthermore, 86 jars from different batches sampled at random from different retailers were tested for their content’s pH which was, in all products, below 4.3.

### 3.4. Case Summary—July 2012

In July 2012, an adult female visited her GP with signs of mild bulbar palsy and blurring of vision. The next day, she deteriorated and was admitted to intensive care with botulism where she received botulinum immunoglobulin three days after admission (five days post-onset). She recovered well and was discharged a few days later.

The patient mentioned that two days before symptom onset, she had dined with friends, consuming a single olive in brine from an Italian artisanal brand purchased at a deli. The other guests, however, avoided eating the olives due to their unusual smell.

Serum collected six days after symptom onset was unsuitable for testing due to delayed collection; however, *C. botulinum* type B was detected in faeces collected on day nine by culture and PCR.

The hosts later provided the opened jar of olives to the hospital and testing at the *C. botulinum* reference laboratory revealed that the pH in the jar was pH 6.65, and BoNT type B by MBA and *C. botulinum* type B by PCR and culture were detected, which confirmed the olives as being the source of intoxication.

The UK food authority was informed and a Rapid Alert System for Food and Feed (RASFF) notification prompted an Italian investigation, revealing that 60 jars from the implicated batch had been sent to a single UK supplier, which distributed them to three delis [12]. The UK Food Authority issued a Food Alert for Action, but no jars from the batch were found, suggesting all had been sold [13]. As a precaution, the suppliers withdrew other batches of the same product. The RASFF report mentioned that the controlling factors, i.e., salt concentration, were found insufficient to prevent growth and toxin production of *C. botulinum* [12].

### 3.5. Case Summary—October 2013

In October 2013 a 40-year-old male Polish national visiting the UK went to a general practitioner for symptoms of food poisoning. He was prescribed azithromycin for gastroenteritis but deteriorated rapidly the same day. He was diagnosed and treated with botulinum antitoxin. His discharge date was not communicated to the Reference laboratory.

The day before symptoms began, he had consumed home-preserved wild mushrooms brought from Poland. Picked three weeks earlier, they were transported in a polythene bag and stored in vinegar, which may have lacked sufficient acidity to prevent *C. botulinum* growth.

Serum collected three days after onset tested positive for botulism by MBA, but due to limited sample volume, confirmation was performed using an ABE polyvalent antiserum, preventing BoNT type identification. Vomit and rectal wash collected six days after onset tested negative by culture and PCR, likely due to late collection.

The family had discarded all traces of any food, including the mushrooms, leaving no samples for testing.

### 3.6. Case Summary—December 2016

A 31-year-old male Italian national was admitted to intensive care in December 2016 with a three-day history of vomiting, followed by blurred vision and classical features of botulism. He was treated with botulinum antitoxin two days after admission, recovered and was discharged a week later.

The patient mentioned that he received home-made tuna in oil that smelled bad and which had been sent by post from Italy. Despite this, he consumed it and vomited 30 min later.

Early serum samples taken at admission were discarded by the hospital laboratory and a serum sample taken two days after admission (five days after symptom onset) tested negative by MBA. However, *C. botulinum* type B and BoNT type B were detected in a faecal sample collected seven days after onset via culture, PCR and MBA, confirming the diagnosis of foodborne botulism in the patient.

The jar of tuna had been opened weeks earlier and stored unrefrigerated in a cupboard. That particular jar was unavailable for testing as it has been discarded, but another similar unopened jar tested negative for *C. botulinum* and BoNT. Still, the pH was measured, and it was pH 5.75 which is within the range for allowing growth of *C. botulinum*.

### 3.7. Case Summary—May 2019

In May 2019, a 28-year-old male was admitted to intensive care with typical neurological clinical symptoms of botulism. Five days earlier, he had brief but severe diarrhoea after eating a pizza, followed three days later by neurological symptoms. Initially treated with immunoglobulin for presumed Guillain-Barré syndrome, he later developed respiratory distress, requiring intubation and ventilation. Botulism was then diagnosed, and he received botulinum antitoxin on day seven after symptom onset. He recovered and was discharged, but the date was not reported to the reference laboratory.

Serum sample collected on admission, i.e., five days after onset, was sent to the reference laboratory, but because of insufficient volume, the clinical diagnosis of botulism by MBA was confirmed via neutralisation with an ABE polyvalent antiserum and the BoNT type could not be determined. Faeces collected seven days after onset tested negative for *C. botulinum* by culture and PCR.

The patient did not wish to submit a full food history, and no pizza remnants were available for testing since all food had been discarded.

### 3.8. Outbreak Summary—September 2023

In September 2023, the French authorities notified the UK of a botulism outbreak linked to contaminated home-made sardines in oil served at a restaurant in southern France. At least 15 people from multiple countries were affected, with 10 hospitalised, 8 requiring intensive care, and 1 fatality [10].

Five British adult male nationals were known to have been exposed. Three developed gastrointestinal and mild neurological symptoms, including muscle fatigue and diplopia, 1 to 3 days after exposure to the sardines. All three were hospitalised and treated with botulinum antitoxin after returning home. All were discharged after brief hospital stays. One other individual remained asymptomatic and did not seek medical care, while another was treated in Spain where he was on holiday.

Serum was collected from the three hospitalised patients, but serum from only one patient, which was negative for BoNT, was sent with sufficient volume to enable testing. *C. botulinum* type B was detected by PCR and cultured from faecal samples from all three patients.

The French reference laboratory confirmed the sardines as the source of C. botulinum neurotoxin type B, with microbiological confirmation in several clinical cases. Local health authorities inspected the restaurant, and all products were recalled [10].

### 3.9. Case Summary—February 2024

In February 2024, a 64-year-old woman developed heartburn, which progressed to vomiting and difficulty swallowing a day after consuming a steak and ale pie at a pub. Over the next three days, she deteriorated and was eventually diagnosed with botulism. She was treated with botulinum antitoxin 4 days after admission, and 8 days after her symptoms first appeared, she recovered and was discharged 5 weeks later.

The serum was tested as it was not known when it has been taken when it was referred to the reference laboratory. When BoNT type B was detected by MBA, the hospital confirmed that the sample had been collected on day 8. *C. botulinum* type B was detected by PCR and isolated from stool samples taken on day 14, both results confirming a diagnosis of botulism.

A food questionnaire revealed that the patient had concerns about the steak and ale pie’s appearance, smell, and taste. However, cooking records at the pub showed that pre-consumption, the food had been cooked for 15 min at 80–82 °C, a temperature sufficient to destroy any BoNT present. She also reported consuming meat pâté on toast at the same pub that day. Within the three days before symptom onset, she purchased duck and chicken meat from a supermarket and ate home-made duck potato bake and spaghetti Bolognese. However, it was unclear from the questionnaire whether she used the purchased meat to prepare these dishes or if they were separate meals. It is unusual in cases of food botulism to have BoNT circulating in the blood 8 days after onset, and without any food remnants available for testing, the source and date of intoxication remained uncertain.

### 3.10. Case Summary—September 2024

In September 2024, a 70-year-old woman became unwell after eating chicken bites snack that she described as “off” the previous day. She initially developed diarrhoea and blurred vision and eventually experienced neurological signs. Initially treated for a stroke, botulism was then rapidly diagnosed, and she was treated with antitoxin on the day of admission.

Stool and serum samples were collected the following day and *C. botulinum* type A was detected by PCR and isolated from the stool, though the serum was not tested for undisclosed reasons.

Because the patient reported that the commercially distributed roasted chicken bites had a foul smell and that she was the only one who consumed them, her husband decided to bring the remnant to the hospital, but unfortunately, the clinician on duty deemed it irrelevant to the case and discarded it, leaving no food available for testing and the source of intoxication unknown.

### 3.11. Statistical Overview

Overall, the 13 cases of botulism in the UK between 2010 and 2024 occurred across seven separate incidents and two distinct outbreaks, with no reported fatalities. Analysis of these incidents reveals that botulinum neurotoxin type B was the predominant type identified, responsible for at least five of the nine confirmed incidents, followed by type A in two incidents and no determined type in two incidents. While the specific food sources were not always confirmed, implicated or suspected sources broadly fell into categories including commercially distributed products (Korma sauce, chicken bites), artisanal (olives, sardines), and home-prepared foods (mushrooms in vinegar and tuna in oil). The data also underscore persistent challenges in achieving microbiological confirmation from clinical specimens or implicated food sources, often due to delay in diagnosing botulism, delayed sample collection, or unavailability of suspected food.

## 4. Discussion

The data presented in this study update the last major UK review by McLauchlin et al. (2006) [6] which examined foodborne botulism over 83 years (1922–2005). While that earlier review documented 62 cases, compared to 13 cases in our 18-year review (2006–2024), the present study highlights several striking differences. Notably, these include the complete absence of fatalities and the rarity and smaller scale of outbreaks in the recent period. This can reflect better clinical diagnosis and the creation of the National Health Service in 1948 which allowed patients to seek help in hospitals or general practitioners free at the point of need, together with improved commercial canning manufacturing processes. The earlier review found that foodborne botulism in the UK was largely linked to home-made or artisanal products, with incidents from the 1990s and 2000s associated with home-prepared imported foods. Our findings confirm this trend with cases linked to imported or artisanal foods such as olives and home-made preserved foods. However, newer cases include a branded korma sauce, possibly pub food, pizza toppings, and a packet of chicken bites, highlighting the potential for contamination in commercial food either after purchase or during food production.

Another notable difference between the historical review and the present study is the proportion of incidents where the food source remained unidentified or unconfirmed by microbiological testing. While food sources were identified in nearly all incidents (all except one) between 1922 and 2005, a higher proportion of cases in the current review only have a suggested or presumed source, with a frequent unavailability of remnants for testing. This discrepancy in source identification may reflect broader challenges impacting public health investigations and laboratory capacity during the more recent period. Factors such as evolving resource allocations within local authorities and healthcare systems, along with the increasing complexity of global food supply chains, could potentially contribute to difficulties in securing timely samples and conducting comprehensive food trace-back investigations. The rapid disposal of implicated foods before public health authorities become involved limits the ability to trace and potentially prevent future cases which is a public health concern that needs addressing. In a similar way and potentially for the same reasons as highlighted above about the lack of food testing, specimen collection is often delayed, leading to reduced detection sensitivity or unavailability. Serum testing frequently yielded negative results due to late sampling or insufficient volume provided, as seen in the 2010 and 2016 cases. Stool testing by PCR and culture appears more reliable as the organism can remain in the host longer than the BoNT in the blood, as experienced by the investigation of seven out of the nine incidents described here compared to four incidents where serum MBA showed a positive result for BoNT. A further concern is the potential worsening of patient conditions due to inappropriate first treatment due to early botulism neurological symptoms being quite similar to other illnesses such as stroke, Guillain–Barre symptoms, and other infectious diseases. The administration of antibiotics in suspected botulism cases can lead to increased toxin release as bacterial lysis occurs in the gut. This underscores the fact that despite botulism being extremely rare in the UK, there is a need for heightened awareness among clinicians regarding appropriate management strategies.

The findings of this review highlight the rarity of foodborne botulism in the UK, with only 13 cases reported between 2010 and 2024. Foodborne botulism incidence is also low in other European countries but higher than in the UK. According to the European Centre for Disease Prevention and Control (ECDC), 84 confirmed botulism cases were reported across the European Union and European Economic Area in 2022, resulting in an overall notification rate of 0.02 cases per 100,000 population. This figure included all types of botulism; however, foodborne botulism was noted as the most common form of the disease frequently caused by inadequately processed, often home-canned, preserved, or fermented foods. In the 2022 ECDC report, the mode of transmission was identified for 35 cases, with 33 (94%) attributed to foodborne sources, primarily caused by BoNT type A and linked to the consumption of canned food, fish, meat, and vegetables. The 2021 ECDC report shows similar trends [14,15]. In comparison, the average annual rate of foodborne botulism in the UK from 2006 to 2024 is approximately 0.001 cases per 100,000 population (or about 1 case per 100 million people per year), and the prevalent type causing intoxication is type B. However, the number of cases is too low to allow any statistical significance or true comparison with the ECDC results. The reasons for the lower UK incidence compared to other European countries are not entirely clear and are unlikely to be due solely to differences in food safety regulations. Instead, they may be more attributable to different food habits, such as a lower prevalence of home-preserved food in the UK, for example, with olives, sardines, tuna or tomatoes, which is more likely to contribute to this lower incidence. Despite this low incidence, the UK is not immune to foodborne botulism risks, and while historically linked to imported or artisanal products, newer cases associated with commercial products like korma sauce, pub food, and chicken bites demonstrate an evolving risk landscape. This necessitates continuous review to adapt clinical and public health risk assessment protocols. This risk is exemplified by the 2011 korma sauce outbreak [11], where it was hypothesised that vacuum loss and pH changes may have allowed *C. botulinum* to proliferate. The absence of cases linked to domestically produced home-canned foods does not eliminate the risk, as globalisation of food supply chains continues to introduce new exposures. The clinical risk assessment for food botulism in the UK used, for historical reasons, to heavily focus on history of consumption of foreign preserved and home-made food; however, the more recent cases, post COVID-19 pandemic, were associated with local food consumption. Therefore, the clinical and public health risk assessment protocols in England have been updated to consider food botulism in a case with the appropriate clinical presentation regardless of local or imported food consumption.

## 5. Conclusions

This review serves to highlight recurring public health challenges in the UK regarding the management of foodborne botulism. Despite remaining uncommon with an overall low case burden, botulism remains a serious public health threat. The absence of fatalities in the UK cases suggests effective clinical management and timely administration of botulinum antitoxin for all cases described here; however, significant diagnostic and public health challenges persist. These include delayed diagnosis and sample collection, inadequate specimens for testing, and the frequent unavailability of suspected food sources, all of which hinder epidemiological investigations. Understanding these challenges in the context of individual cases, even sporadic ones, is crucial for improving public health response and patient outcomes. Improving awareness amongst clinicians to enable them to identify this rare but serious medical emergency, the importance of early sample collection, appropriate treatment strategies, and rapid collection of food history together with food sampling is crucial for improving case outcomes and controlling outbreaks.

## Data Availability

No new data were created or analyzed in this study. Data sharing is not applicable to this article.

## References

[1] Martinez-Carranza M., Skerlova J., Lee P.G., Zhang J., Krc A., Sirohiwal A., Burgin D., Elliott M., Philippe J., Donald S. (2024). Activity of botulinum neurotoxin X and its structure when shielded by a non-toxic non-hemagglutinin protein. Commun. Chem..

[2] Brunt J., Carter A.T., Stringer S.C., Peck M.W. (2018). Identification of a novel botulinum neurotoxin gene cluster in Enterococcus. FEBS Lett..

[3] Rawson A.M., Dempster A.W., Humphreys C.M., Minton N.P. (2023). Pathogenicity and virulence of *Clostridium botulinum*. Virulence.

[4] Erbguth F.J. (2004). Historical notes on botulism, *Clostridium botulinum*, botulinum toxin, and the idea of the therapeutic use of the toxin. Mov. Disord..

[5] Horowitz B.Z. (2011). The ripe olive scare and hotel Loch Maree tragedy: Botulism under glass in the 1920’s. Clin. Toxicol..

[6] McLauchlin J., Grant K.A., Little C.L. (2006). Food-borne botulism in the United Kingdom. J. Public Health.

[7] Solomon H.M., Lilly T. (2001). Clostridium botulinum in Bacteriological Analytical Manual.

[8] Akbulut D., Grant K.A., McLauchlin J. (2004). Development and application of Real-Time PCR assays to detect fragments of the *Clostridium botulinum* types A, B, and E neurotoxin genes for investigation of human foodborne and infant botulism. Foodborne Pathog. Dis..

[9] Grant K.A., Nwarfor I., Mpamugo O., Mithani V., Lister P., Dixon G., Nixon G., Planche T., Courtney M., Morgan J. (2009). Report of two unlinked cases of infant botulism in the UK in October 2007. J. Med. Microbiol..

[10] Meurice L., Filleul L., Fischer A., Burbaud A., Delvallez G., Diancourt L., Belichon S., Clouzeau B., Malvy D., Oliva-Labadie M. (2023). Foodborne botulism outbreak involving different nationalities during the Rugby World Cup: Critical role of credit card data and rapid international cooperation, France, September 2023. Eurosurveillance.

[11] Browning L.M., Prempeh H., Little C., Houston C., Grant K., Cowden J.M., United Kingdom Botulism Incident Management T. (2011). An outbreak of food-borne botulism in Scotland, United Kingdom, November 2011. Eurosurveillance.

[12] Directorate General for Health and Consummers (European Commission) (2013). The Rapid Alert System for Food and Feed 2012 Annual Report.

[13] Food Standards Agency Update on Recall of Divini Di Chicco Olives. The National Archives. http://www.food.gov.uk/enforcement/alerts/2012/jul/olivesupdate.

[14] European Centre for Disease Prevention and Control (2023). Botulism. ECDC. Annual Epidemiological Report for 2021.

[15] European Centre for Disease Prevention and Control (2024). Botulism. ECDC. Annual Epidemiological Report for 2022.

